# Establishment of a chinchilla model of *Achromobacter xylosoxidans*-induced otitis media

**DOI:** 10.1128/spectrum.00985-26

**Published:** 2026-07-06

**Authors:** P. Khampang, K. L. Brockman

**Affiliations:** 1 Department of Microbiology and Immunology, Medical College of Wisconsin 735651, Milwaukee, Wisconsin, USA; 2 Department of Otolaryngology and Communication Sciences, Medical College of Wisconsin 5506, Milwaukee, Wisconsin, USA; Instituto Nacional de Tecnologia Agropecuaria, Buenos Aires, Argentina

**Keywords:** *achromobacter xylosoxidans*, otitis media, chinchilla, experimental model, middle ear infection, bacterial persistence, disease severity, mucosal infection

## Abstract

*Achromobacter xylosoxidans* (Ax) is an emerging opportunistic pathogen associated with chronic respiratory infections, particularly in individuals with cystic fibrosis; however, its role in otitis media (OM) and the mechanisms that underlie middle ear infection remain poorly defined. Despite its initial isolation from middle ear effusions decades ago, no experimental models of Ax-induced OM have been established. Here, we report the development of a reproducible chinchilla model of Ax-induced OM, which, to our knowledge, represents the first experimental model of Ax-induced middle ear infection. Following transbullar inoculation, two well-characterized clinical isolates established middle ear infection and induced measurable changes in tympanometry and otoscopic pathology. Notably, strain-specific differences in disease severity and persistence were observed, with one isolate inducing more pronounced inflammation, reduced tympanic membrane compliance, and prolonged bacterial survival within the middle ear mucosa. At the study endpoint, viable bacteria were recovered from a subset of infected ears despite resolution of overt clinical signs, which indicated a capacity for persistent mucosal infection. These findings demonstrate that Ax can establish and maintain infection within the middle ear and that clinical isolates exhibit distinct pathogenic phenotypes in this model. Together, this work establishes a reproducible and clinically relevant *in vivo* model for the study of Ax-induced OM and provides a foundation for future investigations into host-pathogen interactions, persistence mechanisms, and therapeutic interventions.

**IMPORTANCE:**

*Achromobacter xylosoxidans* is an emerging opportunistic pathogen increasingly associated with chronic mucosal infection, yet its role in otitis media remains poorly defined. Mechanistic studies of middle ear infection have been limited by the absence of an experimental model. We establish the first reproducible animal model of *A. xylosoxidans*-induced otitis media and demonstrate that clinical isolates differ in their capacity to drive disease severity within the middle ear. By enabling controlled investigation of infection dynamics *in vivo*, this model provides a framework to define determinants of pathogenesis and directly compare strain-specific phenotypes across mucosal environments. These findings establish a foundation for future studies of *A. xylosoxidans* infection and therapeutic intervention.

## INTRODUCTION

*Achromobacter xylosoxidans* (Ax) has emerged as an increasingly recognized opportunistic pathogen associated with multidrug resistance and persistent infection. First described in 1971 in patients with chronic otitis media (OM) (1), Ax is a gram-negative, aerobic, non-fermenting bacterium widely distributed in soil and water. Its substantial metabolic versatility and intrinsic resistance to multiple antibiotics and some disinfectants facilitate survival in healthcare-associated environments, including water systems and abiotic surfaces (2–7).

Although infections in immunocompetent individuals are uncommon, Ax has become increasingly prevalent in immunocompromised populations, particularly individuals with cystic fibrosis (CF), the elderly, and patients requiring indwelling medical devices (8–11). Environmental acquisition is believed to be the primary route of transmission; however, patient-to-patient spread has been documented in CF treatment centers (12). Advances in CF care, including improved infection control practices and the introduction of CFTR modulators, have altered the epidemiology of airway infection in some settings (13, 14), yet Ax remains a persistent colonizer in susceptible hosts.

Clinically, Ax most commonly presents as chronic respiratory infection, including ventilator-associated pneumonia and bronchiectasis-associated disease, and is associated with otitis media. In severe or uncontrolled cases, infection may progress to bacteremia, sepsis, and endocarditis (15–18). The increasing incidence of Ax infections, combined with its intrinsic antibiotic resistance and environmental resilience, underscores its growing clinical significance.

We recently established a murine model of acute Ax-induced pneumonia to evaluate the pathogenic potential of clinical isolates obtained from distinct anatomical sites. In this system, both a CF lung isolate (GN008) and a middle ear effusion (MEF) isolate (GN050) reproducibly induced acute pulmonary infection, with strain-dependent differences in disease severity and systemic dissemination (19). These findings demonstrated that clinical Ax isolates retain pathogenic capacity across host niches and highlighted the need to examine Ax infection in additional clinically relevant anatomical sites.

Although Ax was first described in patients with chronic otitis media, experimental systems to study Ax infection within the middle ear have not been established. As a result, the pathogenesis, persistence, and host response to Ax in this niche remain poorly defined. To address this gap, we developed and characterized a chinchilla model of Ax-induced OM. The chinchilla is the gold standard for experimental OM due to its anatomical similarity to the human middle ear and its extensive validation in studies of established otopathogens, including *Haemophilus influenzae*, *Streptococcus pneumoniae*, *Moraxella catarrhalis*, *Pseudomonas aeruginosa*, and polymicrobial or viral-bacterial co-infection models (20–24). Moreover, the model has been widely used in preclinical vaccine development and therapeutic evaluation, making it a highly translational platform for studying Ax infection in the context of middle ear disease (25–27).

Here, we describe the development and initial characterization of a reproducible chinchilla model of Ax-induced OM. Using two clinical isolates derived from human infection, we demonstrate that Ax establishes middle ear infection with features consistent with experimental OM and that isolates differ in disease severity and persistence within this niche.

## MATERIALS AND METHODS

### Bacterial culture

Clinical isolates of *Achromobacter xylosoxidans* (GN050, middle ear isolate, and GN008, cystic fibrosis lung isolate), previously described (28), were obtained from clinical specimens collected at the Medical College of Wisconsin and Froedtert Hospital. Bacteria were cultured on Luria-Bertani (LB) agar plates at 37°C with 5% CO_2_ for 24 h, followed by incubation at room temperature for an additional 24 h. Bacterial inocula were prepared by resuspending plate-grown bacteria in sterile non-pyrogenic injectable 0.9% saline. Cell density was estimated by optical density at 600 nm (OD_600_) and adjusted to the desired concentration based on a previously established OD-CFU relationship (19, 28). The final inoculum concentration (CFU/mL) was confirmed by serial dilution and plating on LB agar.

### Chinchilla infection model

Adult outbred chinchillas (600–800 g; mixed sex) were obtained from Rauscher Chinchilla Ranch and acclimated prior to study initiation. All animals were confirmed to be free of visible illness and otologic disease by otoscopic examination prior to inoculation.

A total of six chinchillas were used for longitudinal assessment of disease progression. Experiments were conducted across two independent study runs performed at separate times under comparable experimental conditions. On experimental day 0, bilateral transbullar inoculation was performed under general anesthesia (intramuscular ketamine/xylazine), with bacteria delivered directly into the inferior bulla. Animals were divided into two groups (*n* = 3 per group) and inoculated with either GN050 or GN008 at a dose of 750 CFU per ear.

Animals were monitored for 21 days for clinical signs of otitis media. Otoscopy and tympanometry were performed at defined time points to assess disease progression. At the study endpoint (day 21), animals were euthanized, and samples were collected. Bullae were aseptically removed and bisected to expose the middle ear cavity. Middle ear mucosa with any adherent biomass was collected. Gross morphology of the middle ear space was documented by dissecting microscopy.

Middle ear mucosal tissues were homogenized in sterile saline, serially diluted, and plated on LB agar. Plates were incubated at 37°C and monitored for bacterial growth. Colony-forming units (CFUs) were determined to assess total bacterial burden and CFU per mg of tissue; however, as bacterial burdens at the study endpoint approached the limit of quantification (1e3 CFU), results were analyzed as culture positive or culture negative rather than as enumerated CFU values.

### Clinical assessment and tympanometry

Clinical progression of otitis media was monitored longitudinally by otoscopy and tympanometry. Otoscopic examinations were performed at defined time points using a handheld video otoscope (Welch Allyn) to assess tympanic membrane appearance, including opacity, erythema, and presence of middle ear effusion. Tympanometry was performed to quantitatively assess middle ear function, including ear canal volume, middle ear pressure, and tympanic membrane compliance. Measurements were obtained using a handheld tympanometer (Interacoustics Titan) according to the manufacturer’s instructions. Each ear was evaluated independently at each time point. Changes in otoscopic scores and tympanometric parameters were used to assess disease progression over the 21-day study period.

### Statistical analysis

All statistical analyses were performed using GraphPad Prism (version 11.0.0; GraphPad Software). Data were presented as mean ± standard error of the mean (SEM) unless otherwise indicated. Comparisons between two groups were performed using unpaired, two-tailed Student’s *t*-tests or nonparametric equivalents where appropriate. For comparisons involving multiple groups or time points, two-way analysis of variance with appropriate *post hoc* testing was used.

Given the small sample sizes, nonparametric tests were applied where data did not meet assumptions of normality. A *P* value of <0.05 was considered statistically significant.

## RESULTS

### Establishment of an Ax-induced OM model in the chinchilla

Chinchillas were infected via transbullar inoculation with either strain GN008 or GN050 to establish an experimental model of Ax-induced OM. Following infection, animals were monitored longitudinally for disease progression using otoscopy and tympanometry over a 21-day study period.

### Otoscopy demonstrated the development of acute OM following Ax infection

**Fig 1:**
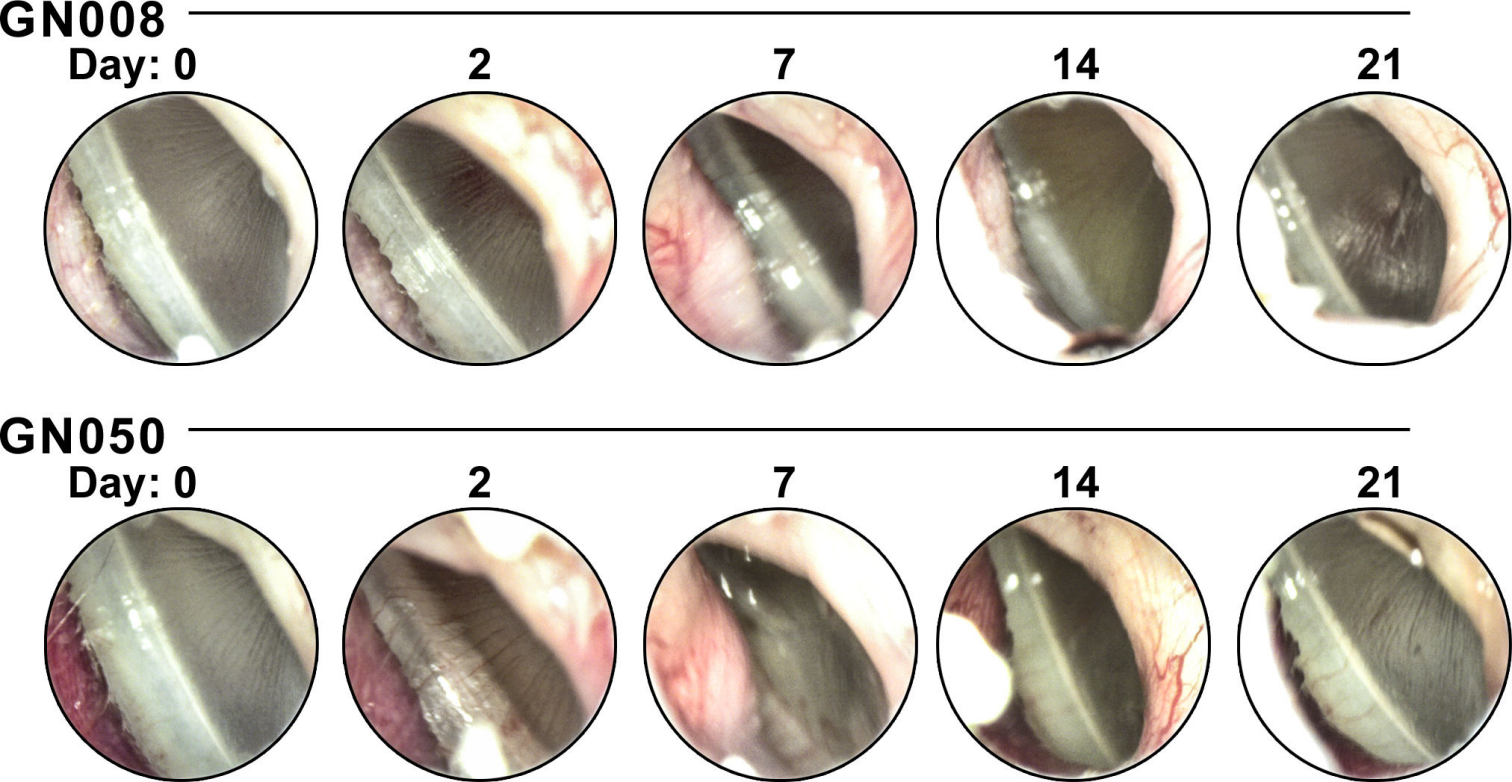
Representative otoscopic images following ME infection with Ax. Serial otoscopic examination of representative chinchilla ears infected with GN008 or GN050. Images are shown at baseline (day 0) and at days 2, 7, 14, and 21 post-challenge. Tympanic membranes remained intact throughout the study. Animals infected with GN050 exhibited increased tympanic membrane opacity and evidence of MEF during peak disease compared with animals infected with GN008.

Otoscopy revealed MEF in all ears by day 2 post-inoculation, which persisted through day 14 in both cohorts. Animals infected with GN050 exhibited more pronounced tympanic membrane opacity and effusion during peak disease. In both cohorts, otoscopic findings resolved by day 21. Representative otoscopic images are shown in Fig. 1.

### Tympanometry reveals greater functional disruption in GN050-infected ears

**Fig 2:**
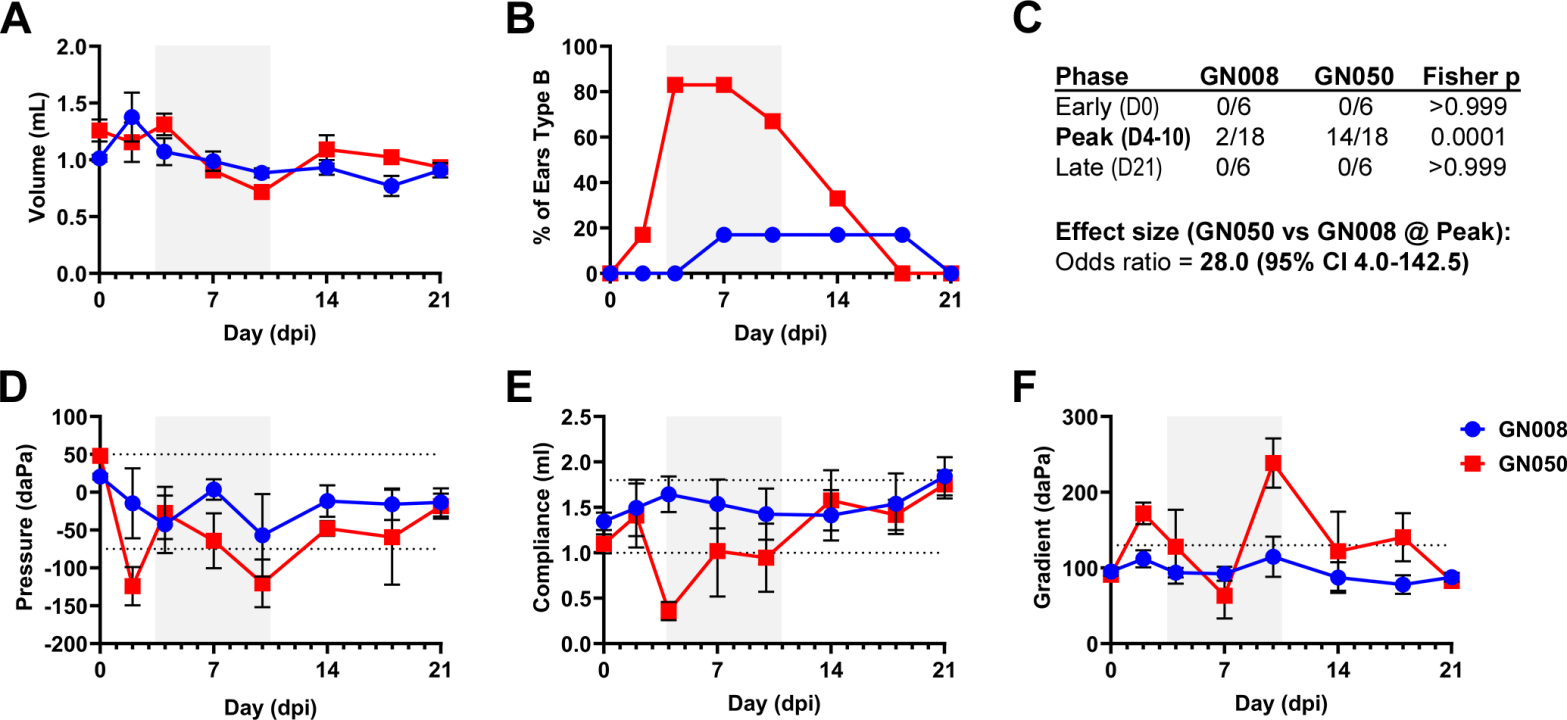
Longitudinal tympanometric changes during experimental otitis media. (**A**) Ear canal volume measured by tympanometry over the course of infection. (**B**) Incidence of Type B tympanograms expressed as the percentage of ears within each cohort at each time point. (**C**) Fisher’s exact test comparing Type B incidence between cohorts during early (day 0), peak (days 4–10), and late (day 21) disease phases. GN050 exhibited significantly greater Type B incidence during the peak disease window compared with GN008 (14/18 vs 2/18 ears; Fisher’s exact test, *P* = 0.0001), corresponding to an odds ratio of 28.0 (95% CI, 4.0–142.5). (**D–F**) Continuous tympanometric parameters, including middle ear pressure (**D**), tympanic membrane compliance (**E**), and tympanometric gradient (**F**). The shaded region indicates the peak disease window (days 4–10) used for phase-based statistical comparisons. Data points represent mean ± SEM. Missing values in panels **D–F** correspond to Type B tympanograms in which no measurable tympanometric peak was detected. Each cohort consisted of six ears (*n* = 6) measured longitudinally across the indicated time points.

To assess functional changes associated with infection, bilateral tympanometry was performed on all animals throughout the 21-day study. Composite tympanograms for each cohort are shown in Fig. S1. Ear canal volumes remained stable for both cohorts across all time points, confirming the absence of tympanic membrane perforation in any animal (Fig. 2A). As expected with this model of outbred mixed-sex animals, disease severity and progression varied between animals and individual ears. Consistent with this variability, individual tympanometric profiles exhibited a broad range of peak compliance and pressure shifts across ears (Fig. S1).

The most pronounced indicator of disease severity was the development of Type B tympanograms. Type B tympanograms reflect severe middle ear effusion and the absence of a measurable tympanometric peak (29) and were strongly associated with GN050 infection. During the peak disease window (days 4–10 post-challenge), 14 of 18 tympanograms from GN050-infected ears exhibited Type B tympanograms compared with only 2 of 18 GN008-infected tympanograms (Fig. 2B and C; Fisher’s exact test, *P* = 0.0001; OR = 28.0, 95% CI, 4.0–142.5).

Consistent with these findings, continuous tympanometric parameters demonstrated greater functional disruption following GN050 infection. Middle ear pressure, an indicator of Eustachian tube dysfunction and fluid accumulation, shifted toward negative values following infection in both cohorts, with GN050-infected ears exhibiting greater negative pressure during the early and peak phases of infection (Fig. 2D). Tympanic membrane compliance and tympanometric gradient measurements likewise indicated more severe disruption in GN050-infected ears compared to those infected with GN008 (Fig. 2E and F). Notably, most GN050-infected ears produced Type B tympanograms during the peak window, which resulted in missing values for these measurements, as these parameters cannot be calculated in the absence of a detectable response curve.

Collectively, these data demonstrated that while both isolates induced OM, disease severity was markedly greater following infection with GN050. In both cohorts, tympanometric parameters indicated that disease peaked between days 4 and 10 and showed clear signs of resolution by day 21, consistent with a reproducible acute otitis media phenotype in this model.

### Clinical observations and bacterial persistence at study endpoint

Clinical complications were uncommon during the course of infection. One GN050-infected animal developed bilateral labyrinthitis on day 3, which resolved by day 9. At the time of dissection (day 21), no visible middle ear fluid was present in any ear. Representative images of dissected bullae at the study endpoint are shown in Fig. S2. To assess bacterial persistence, middle ear mucosa from each ear was collected at the study endpoint (day 21) and plated to detect viable Ax. None of the GN008-infected ears yielded detectable bacteria (zero of six), whereas viable GN050 was cultured from four of six ears. These findings indicate that although clinical signs of OM had resolved by day 21, GN050 was capable of maintaining a detectable mucosal presence, suggesting a capacity for persistent infection within the middle ear (culture-positive ears: mean 1,950 ± 580 CFU per ear).

## DISCUSSION

The establishment of experimental infection models is a prerequisite for the mechanistic investigation of host-pathogen interactions, yet no such system existed for Ax-induced OM despite the organism’s original isolation from middle ear effusions more than five decades ago. The findings reported here address this gap and suggest that clinical Ax isolates are not interchangeable in the middle ear. As Ax has emerged as an important mucosal pathogen, particularly in the cystic fibrosis lung and in systemic infections, experimental systems are needed to examine its pathogenesis across infection sites. This model enables experimental investigation of Ax infection within the middle ear and facilitates comparison with infection at other mucosal sites.

In this study, we utilized two well-characterized clinical isolates of *A. xylosoxidans*, strains GN008 and GN050, which differ substantially in their pathogenic phenotypes (28). GN008 was isolated from the sputum of a patient with cystic fibrosis and has previously been shown to establish persistent infection in experimental models. In contrast, GN050 was isolated from a middle ear effusion and exhibits a more acutely cytotoxic phenotype. In our previously described murine model of acute pneumonia, GN008 induced a comparatively mild disease that was associated with prolonged bacterial persistence within the lung, whereas GN050 caused more severe and rapidly progressing infection that frequently resulted in dissemination and systemic disease (19). These contrasting phenotypes allowed assessment of whether isolate-specific differences in pathogenic potential are similarly reflected during infection of the middle ear and across distinct mucosal environments.

Both Ax strains induced acute otitis media that was consistent with experimental infection models established for other bacterial otopathogens (24, 30, 31). Following direct inoculation into the ME, effusions were readily detected by otoscopic examination within 24–48 h in all animals, indicating reliable and reproducible induction of infection. Notably, these findings were consistent across two independent infection studies performed several months apart, further supporting the robustness of the model despite the relatively small cohort sizes used during early model development. Disease severity peaked between days 4 and 10 post-infection, with GN050-infected ears exhibiting significantly greater clinical signs of pathology. To monitor disease progression over the 21-day study period, we employed otoscopic assessment and tympanometry as established measures of middle ear inflammation and effusion in experimental OM models (29, 32). Consistent with the otoscopic findings, GN050-infected ears exhibited significantly greater ME effusion, which was evident both visually and via reduced tympanic membrane mobility. Despite the presence of substantial effusions and ME pressure, tympanic membrane rupture was not observed in any animals. Preservation of membrane integrity is important for experimental OM models, as perforation can introduce secondary infections and alter effusion drainage. Although not directly assessed in this study, the anatomy of the chinchilla bulla also permits aseptic serial sampling of the ME through the superior bulla, providing an additional advantage for longitudinal studies of infection and host responses (33, 34).

We observed several similarities between the chinchilla OM model and our previously described murine model of acute pneumonia. In both systems, GN050 exhibited a more acutely pathogenic phenotype compared to GN008. Consistent with this observation, GN050, but not GN008, was capable of disseminating from the site of mucosal infection into the bloodstream during the early stages of infection. In the murine pneumonia model, dissemination of GN050 frequently resulted in rapid disease progression and mortality (19). In contrast, although dissemination was observed in the chinchilla OM model, systemic lethality was not observed. This difference is likely influenced by factors such as host size and bacterial inoculum but may also reflect differences in host immune responses and mucosal barrier properties between the lung and middle ear environments.

The contrasting disease phenotypes observed for GN008 and GN050 suggest that individual Ax isolates employ distinct strategies to establish infection within mucosal environments. GN050 induced a more acute inflammatory disease characterized by substantial ME effusion and early dissemination, whereas GN008 produced comparatively milder disease that resolved without detectable bacterial persistence in the ME. These differences are reminiscent of pathogenic strategies observed among established otopathogens, where some organisms induce rapid inflammatory disease, while others establish more persistent infections and evade host clearance. Although the molecular determinants that underlie these phenotypes remain to be defined, the ability to experimentally reproduce isolate-specific disease patterns suggests that intrinsic bacterial factors likely contribute to infection outcomes. Because Ax is capable of biofilm formation, strain-specific differences in aggregate formation or biofilm-like growth may contribute to the distinct disease and persistence phenotypes observed in the ear. Consistent with this possibility, infection with GN050 was associated with visible mucosal biomass within the middle ear, as shown in Fig. S2. However, the present study did not directly assess biofilm architecture, matrix production, or bacterial organization within the mucosa. Future imaging-based studies will be needed to determine whether persistent GN050 infection is associated with organized bacterial aggregates, biofilm-like communities, or other tissue-associated bacterial reservoirs. The chinchilla OM model, therefore, allows investigation of how strain-level variation in Ax influences host-pathogen interactions within the ME.

Despite these similarities, a key difference between infection models was the pattern of bacterial persistence. In the murine lung infection model, GN050 was unable to persist beyond 7 days, whereas GN008 established long-term persistence within the lung for more than 1 month. In contrast, within the ME, GN008 was undetectable by day 21 post-infection, while viable GN050 bacteria remained present in the majority of ears at this time point, even after clinical signs of disease had begun to resolve. These findings suggest that individual Ax isolates may exhibit preferential adaptation to specific anatomical niches, an observation that can be examined across distinct infection models. Analysis of additional clinical isolates will help clarify how niche-specific, rather than simply host-specific, pressures shape Ax adaptation and virulence across mucosal environments.

Consistent with this possibility, preliminary comparison of publicly available complete Ax genomes revealed a large and open pan-genome, supporting substantial genomic diversity within the species. Lung/CF isolates appeared enriched for accessory antimicrobial resistance determinants, including beta-lactamase and aminoglycoside resistance genes, consistent with the strong antibiotic selection pressures associated with chronic airway infection. However, only a single complete ear isolate genome, GN050, is currently available in public repositories, precluding a statistically powered comparison of ear- and lung-associated isolates. Expanded genome sequencing of middle ear and respiratory clinical isolates will, therefore, be needed to determine whether isolate-specific disease phenotypes reflect broader niche-associated genomic signatures.

The present study was designed as an initial characterization of Ax-induced OM rather than a fully powered mechanistic investigation, and cohort sizes reflect the exploratory nature of this work. The relatively small number of animals per group is a limitation inherent to early-stage model development, particularly with chinchillas, where costs and husbandry constraints favor conservative cohort sizes during initial establishment. Importantly, key findings, such as strain-specific differences in disease severity and bacterial persistence, were consistent across two independent infection studies conducted several months apart, providing confidence in their reproducibility despite the conservative cohort sizes.

These studies expand the range of bacterial pathogens that can be experimentally studied in the chinchilla OM model. The most extensively characterized chinchilla models of otitis media involve *Haemophilus influenzae* (particularly non-typeable strains, NTHi), *Streptococcus pneumoniae*, and *Moraxella catarrhalis* (20, 24, 35). The temporal progression and overall disease characteristics observed in the Ax model most closely resemble pneumococcal infection models, suggesting that Ax infection may similarly involve rapid inflammatory responses within the ME (30). In contrast, NTHi infection models typically exhibit a slower disease course with delayed peak pathology and the formation of persistent mucosal biomass (21). Additional chinchilla models have been described using organisms such as *Pseudomonas aeruginosa*, *Staphylococcus aureus*, and *Alloiococcus otitidis*, although these pathogens are more commonly associated with secondary or opportunistic infections (25). While the present study utilized a monoculture infection model, microbial interactions are central to the pathogenesis of most mucosal infections, including OM. Future studies that incorporate polymicrobial infection or viral co-infection will help clarify how Ax behaves within the broader microbial community and whether interactions with established otopathogens alter disease severity, persistence, or dissemination.

The ability of Ax to disseminate from mucosal infection sites into the bloodstream represents an important and clinically significant aspect of its pathogenesis, particularly in immunocompromised individuals (36). The chinchilla model of GN050-induced OM enables the study of bacterial dissemination in the context of a second mucosal infection site and host species. When combined with our previously established murine pneumonia model, these systems create complementary experimental platforms to investigate the bacterial and host factors that contribute to dissemination. Together, these models will allow investigation of determinants of systemic spread, the influence of host niche and site of isolation on dissemination capacity, and the contribution of defined bacterial mutations to tissue-specific disease, persistence, and dissemination. This will enable future mechanistic studies to test whether bacterial factors previously associated with cytotoxicity or persistence in respiratory models play similar or distinct roles during middle ear infection.

Together, this work establishes the first experimental model of Ax-induced otitis media and demonstrates that clinical isolates exhibit distinct pathogenic phenotypes within the middle ear. By combining this chinchilla OM model with previously established models of Ax lung infection, we now have complementary experimental systems that enable investigation of strain-specific virulence, niche adaptation, and mechanisms of bacterial dissemination across distinct mucosal environments. These experimental platforms provide new opportunities to define the bacterial and host factors that govern Ax infection and persistence and will facilitate the development and evaluation of therapeutic strategies that target this emerging pathogen.

## SUPPLEMENTAL MATERIAL

Fig. S1 spectrum.00985-26-s0001.pdf Composite tympanograms from all ears over the course of infection.

Fig. S2 spectrum.00985-26-s0002.pdf Representative gross morphology of the middle ear at study endpoint.

ASM does not own the copyrights to Supplemental Material that may be linked to, or accessed through, an article. The authors have granted ASM a non-exclusive, world-wide license to publish the Supplemental Material files. Please contact the corresponding author directly for reuse.
