# Supplementary material for: Establishment of a chinchilla model of Achromobacter xylosoxidans-induced otitis media
Source: Microbiol Spectr. 2026 Jul 6;14(8):e00985-26. doi: 10.1128/spectrum.00985-26 (PMC13436351; doi:10.1128/spectrum.00985-26)
Supplement: Supplementary file 1 [file spectrum.00985-26-s0001.pdf]

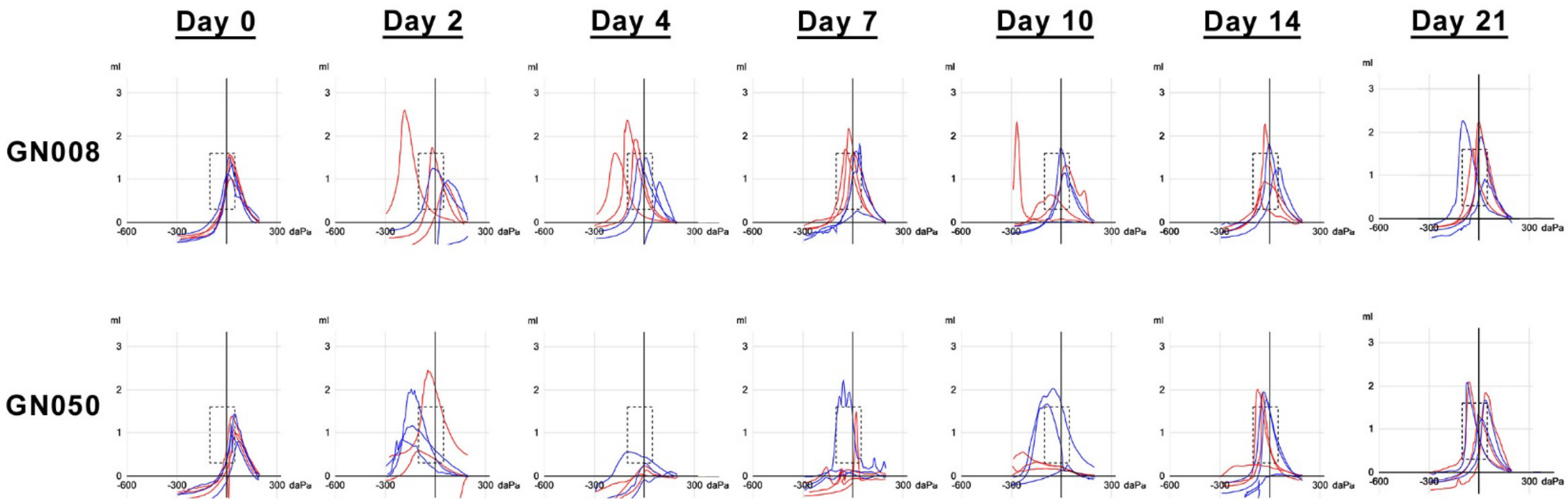

Supplemental Figure S1. Composite tympanograms from all ears over the course of infection. Tympanometry was performed longitudinally following experimental infection with GN008 or GN050. Shown are the raw tympanograms exported directly from the clinical tympanometry system for each ear at the indicated timepoints (days post-infection). Red traces represent right ears and blue traces represent left ears. The vertical line denotes 0 daPa middle ear pressure. The dashed box indicates the instrument-defined normal range of tympanic membrane compliance and pressure. Plot appearance (color scheme, scaling, and reference markers) reflects manufacturer default output parameters. No smoothing or averaging was applied. These composite plots illustrate the distribution and variability of individual ear responses underlying the quantitative analyses presented in Figure 2.
