# Supplementary material for: Establishment of a chinchilla model of Achromobacter xylosoxidans-induced otitis media
Source: Microbiol Spectr. 2026 Jul 6;14(8):e00985-26. doi: 10.1128/spectrum.00985-26 (PMC13436351; doi:10.1128/spectrum.00985-26)
Supplement: Supplementary file 2 [file spectrum.00985-26-s0002.pdf]

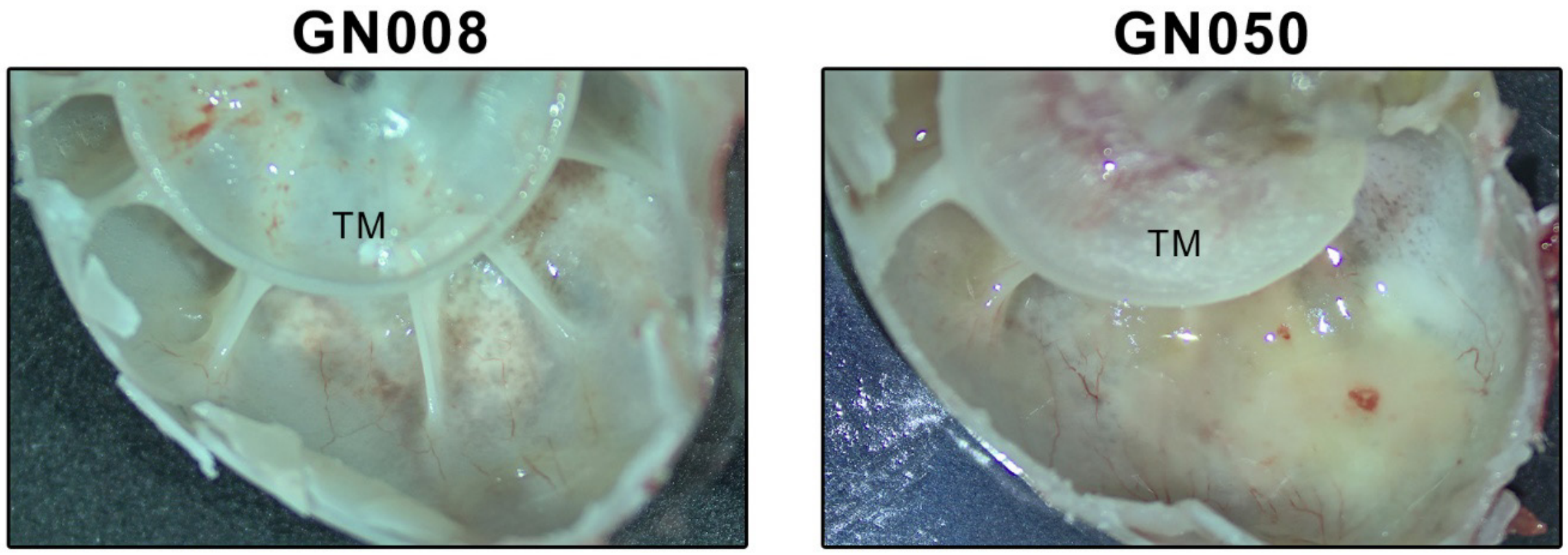

Supplemental Figure S2. Representative gross morphology of the middle ear at study endpoint. Representative images of bisected bullae from chinchillas at day 21 following infection with GN008 or GN050. The tympanic membrane (TM) is indicated for orientation.
